# Renal replacement therapy with net fluid removal lowers intra-abdominal pressure and volumetric indices in critically ill patients

**DOI:** 10.1186/2110-5820-2-S1-S20

**Published:** 2012-12-20

**Authors:** Inneke De laet, Dries Deeren, Karen Schoonheydt, Niels Van Regenmortel, Hilde Dits, Manu LNG Malbrain

**Affiliations:** 1 Department of Intensive Care, Ziekenhuisnetwerk Antwerpen (ZNA) Stuivenberg, Lange Beeldekensstraat 267, Antwerp, B-2060, Belgium

## Abstract

**Background:**

Little is known about the effects of renal replacement therapy (RRT) with fluid removal on intra-abdominal pressure (IAP). The global end-diastolic volume index (GEDVI) and extravascular lung water index (EVLWI) can easily be measured bedside by transpulmonary thermodilution (TPTD). The aim of this study is to evaluate the changes in IAP, GEDVI and EVLWI in critically ill patients receiving slow extended daily dialysis (SLEDD) or continuous venovenous haemofiltration (CVVH) with the intention of net fluid removal.

**Methods:**

We performed a retrospective cohort study in ICU patients who were treated with SLEDD or CVVH and in whom IAP was also measured, and RRT sessions were excluded when the dose of vasoactive medication needed to be changed between the pre- and post-dialysis TPTD measurements and when net fluid loss did not exceed 500 ml. The TPTD measurements were performed within 2 h before and after SLEDD; in case of CVVH, before and after an interval of 12 h.

**Results:**

We studied 25 consecutive dialysis sessions in nine patients with acute renal failure and cardiogenic or non-cardiogenic pulmonary oedema. The GEDVI and EVLWI values before dialysis were 877 ml/m² and 14 ml/kg, respectively. Average net ultrafiltration per session was 3.6 l, with a net fluid loss 1.9 l. The GEDVI decreased significantly during dialysis, but not more than 47.8 ml/m² (*p *= 0.008), as also did the EVLWI with 1 ml/kg (*p *= 0.03). The IAP decreased significantly from 12 to 10.5 mmHg (*p *< 0.0001).

**Conclusions:**

Net fluid removal by SLEDD or CVVH in the range observed in this study decreased IAP, GEDVI and EVLWI in critically ill patients although EVLWI reduction was modest.

## Background

Renal replacement therapy (RRT) is frequently used in the ICU for patients with acute renal failure, fluid overload, refractory metabolic acidosis or intoxication. Several authors have studied the effects of different modalities of RRT on systolic and diastolic left and right ventricular functions in patients with chronic renal failure or congestive heart failure [1,2], but studies on changes in cardiac preload and extravascular lung water index (EVLWI) in critically ill patients are scarce [3-5].

Patients with increased intra-abdominal pressure (IAP) above 12 mmHg, or thus intra-abdominal hypertension (IAH), pose a special problem in the ICU with increased morbidity and mortality [6]. Whereas decompressive laparotomy is the definite treatment, recent data suggest that medical treatment options should be attempted first [7]. Because of the nature of the illness and injury associated with IAH or abdominal compartment syndrome (ACS), these patients retain large volumes of sodium and water, and due to capillary leak, this will exacerbate tissue oedema and third spacing triggering a vicious cycle of ongoing IAH. This is exacerbated by excessive crystalloid resuscitation. In the early stages of IAH, diuretic therapy can be considered to mobilise the oedema, but only if the patient is haemodynamically stable [8,9]. Many patients, however, will not respond to diuretics or even develop anuria as renal blood flow is reduced due to IAH [10]. In these cases, the institution of RRT with fluid removal by intermittent dialysis or continuous venovenous haemofiltration (CVVH) should be considered [11-15].

The global end-diastolic volume index (GEDVI) and EVLWI can easily be measured bedside by transpulmonary thermodilution (TPTD). Although concern has recently been expressed that GEDVI may substantially overestimate the sum of the volumes of the four heart chambers at end-diastole [16], it has been extensively validated as a marker of cardiac preload, especially in IAH [17-20].

The primary aim of this study was to evaluate the possible changes in IAP, GEDVI and EVLWI in critically ill patients receiving slow extended daily dialysis (SLEDD) or CVVH with the intention of net fluid removal. In a *post hoc *analysis, we evaluated whether ultrafiltration decreased EVLWI without excessive compromise of cardiac output.

## Methods

### Study design, setting and participants

We performed a retrospective cohort study at the 24-bed medical and surgical ICU of the ZNA Stuivenberg General Hospital in Antwerp, Belgium. The study was conducted in accordance with the study protocol, the Declaration of Helsinki and applicable regulatory requirements. The local Institutional Review Board and Ethics Committee approved the protocol. In view of the retrospective nature of the study, which did not demand a deviation from standard clinical ICU care, informed consent from the patient or the next of kin was not required. All ICU patients who were treated with RRT and who received concomitant IAP and minimal invasive haemodynamic monitoring with TPTD were eligible for the study. The RRT sessions were excluded when the dose of vasoactive medication needed to be changed between the pre- and post-dialysis TPTD measurements and when net fluid loss did not exceed 500 ml.

### Study protocol and materials

#### Renal replacement therapy

Venous access was obtained via a coaxial double lumen catheter of 14 French in the internal jugular or femoral vein, not adjacent to the PiCCO catheter to avoid distortion of the thermodilution curve. CVVH was performed using the Aquarius haemofiltration circuit (Edwards Lifesciences, Irvine, CA, USA) with a polyethersulfone membrane of 1.9 m^2^, type Aquamax (Edwards Lifesciences, Irvine, CA, USA). Blood pump rate was 150 ml/min with a substitution rate of 1 l/h of bicarbonate-buffered Hemosol solution (Hospal, Lyon, France). SLEDD was performed using the Cobe Centrysystem 3 (Gambro, Inc., Lakewood, CO, USA) with a blood pump rate of 300 ml/min and a dialysate flow of 500 ml/h. Biocompatible membranes and bicarbonate dialysate were standard. Duration per session was 6 h. Anticoagulation for both techniques was performed with low molecular weight heparin. CVVH was chosen for haemodynamically unstable patients, while the others received diurnal SLEDD. Four patients received only SLEDD; two, only CVVH, while the others received both therapies during their stay.

The choice of desired net ultrafiltration and ultrafiltration rate was based on haemodynamic and respiratory variables, and cumulative fluid balance. In case of hypotension, colloids (mostly albumin 20%) were administered, and the ultrafiltration rate was decreased. The dosage of inotropes and vasopressors was kept constant during dialysis, as were the ventilator settings. If the patient's condition did require a change in vasopressor dose, the session was excluded from analysis.

The net fluid loss was calculated by subtracting the total output (including insensible water loss) from the total input. Insensible water loss was calculated with the formula of Dubois, where body surface area = 71.84 × (body weight in kilograms) ^0.425 ^× (height in centimetres) ^0.725^. In case of mechanical ventilation or active humidification, this value was divided by two. For each body temperature increase of 1°C above 37°C, a 13% increase in insensible water loss was calculated [21]. Net ultrafiltration was defined as the absolute value of the difference between the volume of dialysis solution used during dialysis and the volume drained at the end of the session.

#### Transpulmonary thermodilution measurements

Apart from the dialysis catheter, each patient had a central venous catheter, which is not adjacent to the thermistor-tipped thermodilution catheter to avoid the cross-talk phenomenon [22]. Single TPTD measurements were obtained by central venous injection of 20 ml of 'iced' (<8°C) normal 0.9% saline and carried out by ICU nurses who were not aware of the study purpose. In each patient, a set (three injections) of thermodilution determinations was performed within 2 h before and a set within 2 h after SLEDD, in the same body position. In case of CVVH, thermodilution measurements were performed in the morning and in the evening with an interval of 12 h. For each set of thermodilution determinations, the calculated mean values of the determinations were used for haemodynamic management and statistical analysis. The cardiac output (CO) was determined by TPTD using the Stewart-Hamilton method [23,24]. Calculations were carried out with the following equations using a computer system (PiCCOplus, Pulsion Medical Systems, Munich, Germany) [24]. The intrathoracic thermal volume (ITTV) and pulmonary thermal volume (PTV) were respectively calculated from the mean transit time (MTt), and the exponential downslope time (DSt) of the thermodilution curve: ITTV = CO × MTt and PTV = CO × DSt. Theoretically, ITTV consists of the PTV and the sum of the end-diastolic volumes of all cardiac chambers. Accordingly, global end-diastolic volume (GEDV) was calculated as follows: GEDV = ITTV − PTV. Based on a linear relation between GEDV and intrathoracic blood volume (ITBV), ITBV = 1.25 × GEDV. Extravascular lung water (EVLW) is the difference between ITTV and ITBV. Absolute values for GEDV and EVLW were normalised as indexed by body surface area (GEDVI) and body weight (EVLWI). The difference between GEDVI before and after dialysis was called ΔGEDVI (ΔGEDVI = GEDVI_post _− GEDVI_pre_). Accordingly, ΔEVLWI = EVLWI_post _− EVLWI_pre_.

#### Intra-abdominal pressure

The IAP was measured using the FoleyManometer (Holtech Medical, Charlottenlund, Denmark) as described elsewhere [25]. Briefly, a special urinary drainage tubing fitted with a bio-filter was inserted between the Foley catheter and the urine drainage bag. The IAP was measured as the height of the meniscus of the urine column. With this technique, a maximal amount of 20 ml is used for priming, and the mid-axillary line at the level of the iliac crest was used as zero reference. The FoleyManometer is scaled in increments of 0.5 mmHg. The difference between IAP before and after dialysis was called ΔIAP (ΔIAP = IAP_post _− IAP_pre_).

### Analysis

Values are expressed as mean with standard deviation if normally distributed. In view of the small sample size, we also compared the median IAP, GEDVI and EVLWI of each patient before dialysis with the corresponding median IAP, GEDVI and EVLWI after dialysis. Since we were concerned that the differences between the two sets of observations might not be normally distributed, we used the Wilcoxon matched pairs signed-rank sum test.

Because of repeated measurements in each patient, we used weighted analysis, as described by Bland and Altman [26,27] to investigate correlations. A *p *value < 0.05 was considered significant. Calculations were performed with SPSS (version 17.0.1; SPSS, Chicago, IL, USA) in combination with the statistical tables from Vassar College for calculation of *p *in the weighted analysis [28].

## Results and discussion

### Results

#### Patients

We studied nine patients (four women, five men), admitted to the ICU because of primary diagnoses or a combination of acute cardiogenic oedema (*n *= 5), sepsis (*n *= 5), pneumonia (*n *= 5) and chronic obstructive pulmonary disease (*n *= 1), who received RRT because of acute renal failure. Median age was 69 years, and median APACHE-II score was 22.1. All except one patient were mechanically ventilated, and all except one patient received vasopressors and/or inotropes. All patients had pulmonary oedema according to the definition of Mitchell and colleagues (EVLWI greater than 7 ml/kg) [29]. Three of the patients had pure cardiogenic pulmonary oedema, while six had non-cardiogenic pulmonary oedema (acute lung injury (ALI) or acute respiratory distress syndrome (ARDS)). In two patients of the latter group, increased filling pressures (central venous pressure above 18 mmHg) and GEDVI developed after the formation of non-cardiogenic pulmonary oedema, hence creating a combination of cardiogenic and non-cardiogenic pulmonary oedema. RRT was well tolerated, and major haemodynamic parameters did not change significantly (Table 1). The median EVLWI before the dialysis session was 14 ml/kg and ranged from 7 to 29 ml/kg, and the GEDVI before dialysis was 878 ml/ m² and ranged from 662 to 1,250 ml/m². The median IAP before the dialysis was 12 mmHg and ranged from 6 to 17.5 mmHg.

**Table 1:** Haemodynamic parameters before and after renal replacement therapy

	Before	After	*p *value
CO (l/min)	7.1 ± 1.2	6.9 ± 1.6	NS
CI (l/min·m^2^)	4 ± 0.7	3.8 ± 0.9	NS
MAP (mmHg)	85.4 ± 13.1	81.5 ± 20.4	NS
PPV (%)	8.1 ± 7.3	8.6 ± 6.8	NS

CI, cardiac index; CO, cardiac output; MAP, mean arterial pressure; PPV, pulse pressure variation.

#### Dialysis

The data on 25 consecutive dialysis sessions were collected (range one to six per patient). The average net ultrafiltration per dialysis session in each patient was 3.6 l and ranged from 2.0 to 7.5 l, and the average net fluid loss was 1.9 l and ranged from 0.6 to 3.6 l.

#### Changes in IAP

**Figure 1:**
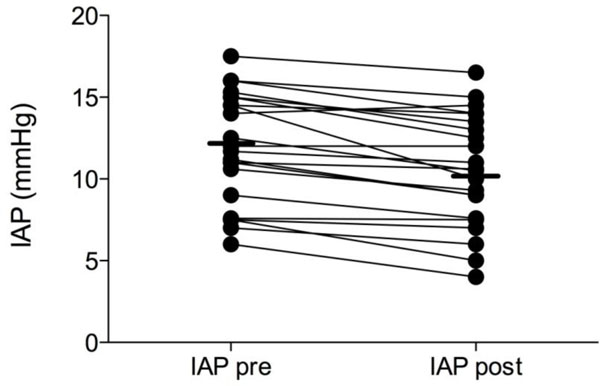
**Effect of renal replacement therapy with net fluid removal on IAP (millimetres of mercury)**. Presented as an individual patient data plot before (pre) and after (post) RRT.

**Figure 2:**
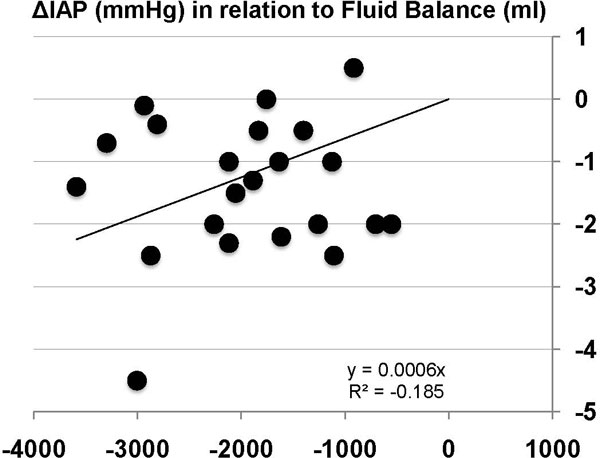
**Regression plot of negative fluid balance amount and ΔIAP**. Regression plot showing the amount of negative fluid balance (millilitres) on the X-axis obtained during RRT in relation to changes in intra-abdominal pressure (ΔIAP, millimetres of mercury) on the Y-axis.

The median IAP after dialysis was 10.5 mmHg (range 4 to 16.1 mmHg). The IAP decreased significantly during dialysis (*p *< 0.0001). The median ΔIAP per dialysis session was −1.4 mmHg and ranged from −4.5 to +0.5 mmHg. Figure 1 shows the effect of RRT on individual IAP values. The more negative the fluid balance, the greater the reduction in IAP (Figure 2).

#### Changes in GEDVI

**Figure 3:**
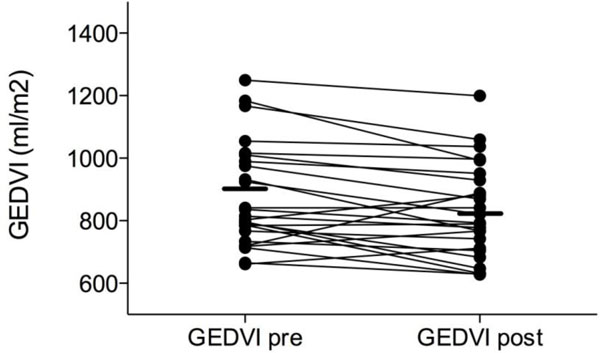
**Effect of renal replacement therapy with net fluid removal on GEDVI (millilitres per square metre)**. Presented as an individual patient data plot before (pre) and after (post) RRT.

The GEDVI after dialysis was 830 ml/m² (range 628 to 1,199 ml/m²). GEDVI decreased significantly during dialysis (*p *= 0.008). The median ΔGEDVI per dialysis session was −47.8 ml/ m² and ranged from −191 to +170 ml/m² (with an absolute median decrease per patient of 55.3 ml/m²). Figure 3 shows the effect of RRT on individual GEDVI values. For the correlation between net fluid loss and ΔGEDVI, the weighted correlation coefficient was *r *= 0.137, *p *= 0.7. For the correlation between GEDVI_pre _and ΔGEDVI, the weighted correlation coefficient was *r *= −0.479, *p *= 0.1.

#### Changes in EVLWI

**Figure 4:**
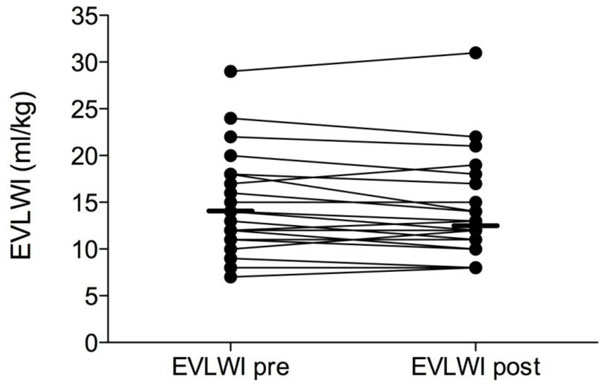
**Effect of renal replacement therapy with net fluid removal on EVLWI (millilitres per kilogram)**. Presented as an individual patient data plot before (pre) and after (post) RRT.

The median EVLWI after dialysis was 13 ml/kg (range 8 to 31 ml/kg). EVLWI decreased significantly during dialysis (*p *= 0.05). The median ΔEVLWI per dialysis session was −1 ml/kg and ranged from −4 to +3 ml/kg, but apart from the extreme case with +3 ml/kg, EVLW decreased or remained the same in all patients (with an absolute median decrease per patient of 49.3 ml). Figure 4 shows the effect of RRT on individual EVLWI values. For the correlation between net fluid loss and ΔEVLWI, the weighted correlation coefficient was *r *= −0.468, *p *= 0.1. For the correlation between EVLWI_pre _and ΔEVLWI, the weighted correlation coefficient was *r *= −0.167, *p *= 0.7.

## Discussion

The main finding of this study is that net ultrafiltration decreased EVLWI by a small but statistically significant amount in this sample of critically ill patients, without excessively reducing cardiac preload, as measured by GEDVI, and this also resulted in a modest but significant decrease in IAP.

RRT with fluid removal has been recommended by the World Society of the Abdominal Compartment Syndrome (http://www.wsacs.org) as a non-surgical management strategy for IAH, mainly in patients after excessive crystalloid resuscitation or capillary leak where oedema of the abdominal wall and the gut are contributing factors to the IAH [30]. Concern using these techniques in patients with ongoing inflammatory processes or haemodynamic instability has centred on the question whether fluid removal by RRT may lead to intravascular hypovolaemia and adverse haemodynamic effects. This study was designed as an observational study to document the effect of fluid removal by RRT on IAP and EVLWI (as outcome measures, a decrease of both parameters being the desired effect) and GEDVI (as a measure of preload to assess adverse effects).

To our knowledge, this is the first study that demonstrates the use of TPTD in the context of dialysis in critically ill patients. Use of this method deserves special interest, because it may be used in the future to guide ultrafiltration, based on GEDVI and EVLWI goals. Further studies are needed to investigate whether such ultrafiltration strategies result in more thorough fluid loss, fewer episodes of hypotension or better outcome.

Although the median absolute reduction of 55.3 ml/m² in GEDVI was statistically significant, its clinical relevance is very small. Blood volume variation during dialysis resulted from the equilibration of ultrafiltration rate and vascular refilling rate [31]. In the course of a dialysis session, fluid is withdrawn from the intravascular compartment, and blood volume tends to fall. This transient reduction of blood volume elicits several compensatory mechanisms that generate a vascular refill process from the over-hydrated interstitium [32,33]. In this study, the effect of fluid removal through RRT on cardiac preload was deemed to be acceptable in a clinical situation.

The median EVLWI reduction was very modest, counting approximately only 1 ml/kg or 65 ml for an average net fluid loss of 1.9 l. This reminds of other studies showing a decrease in EVLWI during dialysis in patients receiving long-term intermittent haemodialysis, however without a significant correlation between computed tomography graphic density changes and net ultrafiltration [34]. These results cannot simply be extrapolated to critically ill patients. Indeed, the distribution of extravascular water among different body compartments differs from patient to patient, particularly in critically ill patients with varying degrees of capillary leak. The mobilisation of extravascular water from other regions than the lungs may be the reason for the poor reduction of EVLW and for the fact that our results do not agree with studies in non-critically ill patients with end-stage renal disease. These have demonstrated a reduction in early diastolic filling of the left and right ventricles and a reduction of the left ventricle size after dialysis, possibly indicating a clinically relevant reduction of cardiac preload [1,2]. Furthermore, it is possible that equilibration between intra- and extravascular compartments in patients with capillary leak takes hours after dialysis to be completed and also that ongoing pathophysiologic processes resulted in further leak of fluid in the lungs during dialysis. Not surprisingly, the reduction in EVLWI was correlated, although not significantly, with net fluid loss, suggesting that more fluid loss implicates more EVLW reduction.

While the reducing effect of ultrafiltration on EVLWI in patients with cardiogenic pulmonary oedema is well known [35,36], this effect has been described but is less clear in animal models and patients with ALI or ARDS [36,37]. However, although some results have been conflicting, zero balance continuous haemofiltration may improve cardiopulmonary function and reduce pulmonary oedema in these patients, perhaps by removing pro-inflammatory cytokines [38]. The dialysis dose used in this study was not calculated to allow for significant cytokine removal.

There are some limitations in our study. First, the data are observational, and the numbers of patients studied is small. Second, one might argue that the dialysis procedure itself may interfere with PiCCO measurements although no reliable data on that subject exist [39]. Therefore, PiCCO measurements were performed either both during haemofiltration (CVVH) or before and after dialysis (SLEDD). Third, the analysis did not look at the timing and dosing effect of RRT either early after ICU admission or later during the course of the disease. Fourth, the range of IAP observed in this study was quite low, and it remains to be proven whether the same effects can be observed in patients with IAH or ACS. Furthermore, the study population consists of a mix of patients with cardiogenic pulmonary oedema and ARDS, making it impossible to draw conclusions on either separate group.

Since dialysis sessions where vasopressor or inotrope dose was changed during the dialysis session were excluded, this study population was a selected population that tolerated fluid removal well (since patients not tolerating fluid removal are more likely to have received an increase vasopressor dose). This study should be seen mainly as confirmation that fluid removal during RRT can successfully decrease IAP and EVLWI at least in some patients. To identify prognostic factors to determine which patients might benefit most with this strategy (and present least haemodynamic compromise), a prospective interventional trial including all fluid overloaded patients with IAH (after shock state is resolved) should be performed.

## Conclusions

Ultrafiltration by SLEDD or CVVH in the range observed in this study marginally but significantly decreased extravascular water in a selected population of critically ill patients without a negative impact on cardiac preload. Moreover, this treatment resulted in a modest but significant drop in IAP. Transpulmonary thermodilution deserves special interest in this context because it may be used in the future to guide ultrafiltration. To identify those patients likely to benefit most from this approach, a prospective study including all fluid overloaded patients with IAH (after resolution of the shock state) should be performed.

## Abbreviations

ACS: abdominal compartment syndrome; ALI: acute lung injury; ARDS: acute respiratory distress syndrome; CO: cardiac output; CVVH: continuous venovenous haemofiltration; DSt: exponential downslope time; EVLW(I): extravascular lung water (index); GEDV(I) = global end-diastolic volume (index); IAH: intra-abdominal hypertension; IAP: intra-abdominal pressure; ITBV: intrathoracic blood volume; ITTV: intrathoracic thermal volume; MTt: mean transit time; PTV: pulmonary thermal volume; RRT: renal replacement therapy; SLEDD: slow extended daily dialysis; TPTD: transpulmonary thermodilution.

## Competing interests

MLNGM is a member of the Medical Advisory Board of Pulsion Medical Systems. The other authors have no conflicts of interest.

## Authors' contributions

DD, IDL, NVR, KS, HD and MLNGM planned the study, were responsible for the design, coordination and drafting the manuscript. DD participated in the data collection, performed the statistical analysis and drafted the manuscript. MLNGM and IDL conceived the study and its design, participated in data collection, helped to draft the manuscript and made the final review. All authors read and approved the final manuscript.
